# Phase equilibria among *η*-Fe_2_Al_5_ and its higher-ordered phases

**DOI:** 10.1080/14686996.2021.1915691

**Published:** 2021-05-28

**Authors:** Tetsuya Hamada, Masaya Higashi, Kodai Niitsu, Haruyuki Inui

**Affiliations:** a Department of Materials Science and Engineering, Kyoto University, Kyoto, Japan; b Center for Elements Strategy Initiative for Structure Materials (ESISM), Kyoto University, Kyoto, Japan

**Keywords:** Phase equilibrium, phase diagram, intermetallic compound, superlattice structure, antiphase boundary, aluminized steel, Fe–Al, galvanized steel, plating, 10 Engineering and Structural materials, 106 Metallic materials, 212 Surface and interfaces, 503 TEM, STEM, SEM, 501 Chemical analyses

## Abstract

Phase equilibria among the *η*-Fe_2_Al_5_ phase and its higher-ordered phases with the *η* framework structure were determined experimentally. The solubility range of the *η* phase at elevated temperature does not differ remarkably from that in previous studies, but this phase is found to undergo complicated phase transformations upon cooling. Four phases are present, namely *η*’, *η*”, *η*”’ and *η*^m^, with higher-order atomic orderings in the *c*-axis chain sites of the orthorhombic crystal structure of the parent *η* phase. The *η*” and *η*”’ phases form on the Al-poor and Al-rich sides, respectively, in equilibrium with the *ζ*-FeAl_2_ phase below ~415°C and *θ*-Fe_4_Al_13_ phase below ~405°C. The *η*’ and *η*^m^ phases become stable below 312°C and 343°C with the peritectoid reactions *η*’ → *η*^m^ + *η*”’ and *η*^m^ → *η* + *η*”, respectively. The *η* phase is not stable below 331°C with the eutectoid reaction of *η*^m^ + *η*”’ → *η*. On the basis of these findings, we unraveled the phase equilibria among the *η*-Fe_2_Al_5_ phase and its higher-ordered phases with the *η* framework structure.

## Graphical abstract

**Figure:**
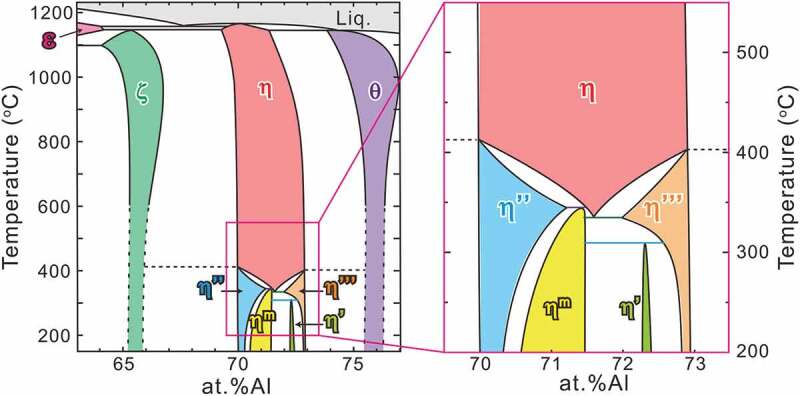

## 1. Introduction

The Fe–Al binary system is one of the basic and important binary systems from which vast practical Fe-based alloys have stemmed. The body-centered cubic solid solutions that are stable over a wide range of the Fe-rich portion have garnered extensive attention in various structural [1–3], functional [4,5], magnetic [6], and spintronic applications [7]. Intermetallic compounds in the Al-rich portion have also played a key role in the surface coating of steels, of which one practical application is hot-dip aluminized and Al-added galvanized steels. Aluminized and galvanized steels with a minor Al addition are used practically as alternatives to galvanized steels because of their superior heat and oxidation resistance [8,9]. The *η* phase (Fe_2_Al_5_) is known to play an essential role in aluminized and Al-added galvanized steels, because the *η* phase is the main constituent phase in the coating layers of aluminized steels and its instantaneous formation on the steel substrate of Al-added galvanized steels suppresses explosive formation (technically termed ‘burst’) of brittle Fe–Zn intermetallic compounds [8–11]. A precise understanding of the phase stability and mechanical properties of the *η* phase is therefore indispensable to better design aluminized and Al-added galvanized steels.

**Figure 1.**
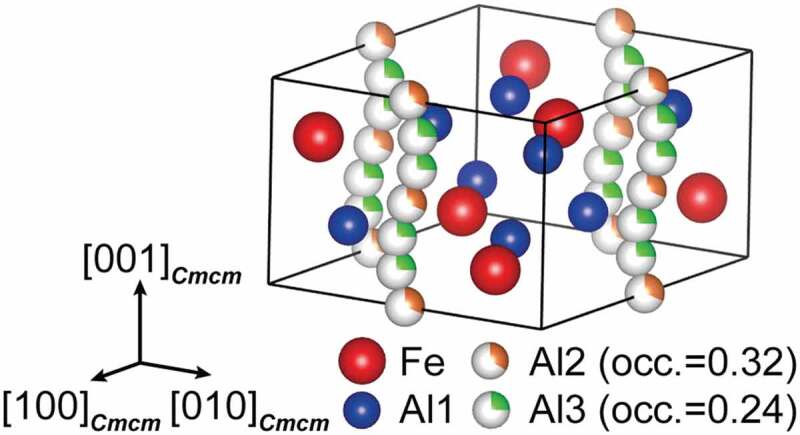
Crystal structure of *η* phase (space group *Cmcm*) reported by Burkhardt [12], which consists of a full occupied framework structure with four Fe and eight Al atoms and partially occupied chains with two Al2 and four Al3 sites along the *c*-axis of the orthorhombic unit cell

According to Burkhardt et al. [12], the crystal structure of the *η* phase (space group: *Cmcm*) is composed of an FeAl_2_ framework of four Fe and eight Al atoms in Fe and Al1 sites with full occupancies and a chain of six Al (two Al2 and four Al3) sites with partial occupancies aligned along the orthorhombic *c*-axis (hereafter called *c*-axis chain), as shown in Figure 1. Initially, it has been considered that only Al atoms occupy the *c*-axis chain so that the relatively large solubility range of the *η* phase (from Fe–69 to –73 at.%Al) is allowed by varying the Al occupancies in the *c*-axis chain sites [13–16]. Becker et al. [17], however, have recently reported from their X-ray diffraction (XRD) study that not only Al but also Fe atoms occupy the *c*-axis chain sites of the *η* phase and that four different phases exist (*η*’, *η*”, *η*”’ and *η*^m^) with higher-order atomic ordering in the *c*-axis chain sites*, as was confirmed experimentally by Okamoto et al. for the *η*’ and *η*” phases [18,19]. The available crystallographic data on the *η* and higher-ordered phases are listed in Table 1 together with neighboring intermetallic phases (*ε, ζ*, and *θ*). Controversy and uncertainty remain regarding the crystallographic structures and solubility ranges of the *η* phase and its higher-ordered phases. Furthermore, despite many reports on the Fe–Al phase equilibria [13–16], the formation reactions of these higher-ordered phases have not been unraveled. Because a thermodynamic assessment of the ternary (or more) systems relies on the constituting binary host systems, a reassessment of the Fe–Al binary phase equilibria provide fundamental and broader impacts on the design guidelines for a vast array of practical Fe-based alloy systems.

**Table 1.** Crystal structures of Al-rich intermetallic phases and identified phases in this study

Phase	Formula	Composition (approx., at.% Al)	Pearson symbol	Space group
*ε*	Fe_5_Al_8_	57–64	*cI*52	*I*$\overset{ˉ}{4}$3 *m* [28]
*ζ*	FeAl_2_	64–67	*aP*19	*P*$\overset{ˉ}{1}$ [29]
*η*	Fe_2_Al_5_	69–73	*oC*24	*Cmcm* [12]
*η*”	Fe_3_Al_7+x_	69–71	*oP*284^a^	*Pmcn*^b^ [19]/*Xmcm* and *Immm*^c^ [17]
*η*^m^	?	71–72	?	*C*2/*m*11 [30]
*η*’	Fe_3_Al_8_	72–73	*mC*44	*C*2/*c* [18,23]
*η*”’	–^c^	72–73	–^c^	*P*2_1_/*c*(0*b*0)00 and *P*2_1_/*c*(0*b*0)*s*0^c^ [17]
*θ*	Fe_4_Al_13_	74–77	*mC*102	*C*2/*m* [31]

^a^For *M* = 19 (19 unit cells of the *η* phase stacked along the *c*-axis direction).

^b^Fifth setting of *Pnma*.

^c^Incommensurately modulated composite crystal structure.

In the present work, we investigated the phases present, their formation reactions, solubility ranges and thermal stabilities by integrating XRD, transmission electron microscopy (TEM), scanning transmission electron microscopy (STEM) and differential scanning calorimetry (DSC). All the results are wrapped up to the phase diagram for the *η* and its higher-ordered phases.

* Footnote:

The four phases (*η*’, *η*”, *η*”’ and *η*^m^) that exhibit higher-order atomic ordering in the *c*-axis chain sites of the *η* phase are hereafter termed ‘higher-ordered’ phases, to distinguish them from the ordered phase of *η*. Because the crystal structures of these four higher-ordered phases are based on that of the *η* phase, the term ‘superlattice’ is used to describe the crystal structures of the higher-ordered phases, whereas ‘parent’ and ‘fundamental’ are used for the *η* phase.

## 2. Experimental procedures

Alloy ingots with compositions listed in Table 2 were prepared from high-purity Al (99.99 wt.%) and Fe (99.99 wt.%) by arc melting under high-purity Ar gas flow. Fabricated ingots were encapsulated in a quartz tube that was backfilled with Ar gas and homogenized at 900°C for 1 day, followed by water quenching by breaking the quartz tube. The ingots were cut into small fragments and equilibrated between 250°C and 1000°C from 0.5 to 60 days followed by water quenching by breaking the quartz tube, respectively. Thin foils for TEM observations were prepared by the focused-ion beam (FIB)-scanning electron microscopy (SEM) (FEI Quanta 3D 200i dual-beam system, Thermo Fisher Scientific, USA) in-situ lift-out technique [20]. TEM and high-angle annular dark-field (HAADF) observations were performed by a 200-kV TEM (JEM-2000FX, JEOL, Japan) and STEM (JEM-ARM200F, JEOL, Japan). Some specimens for HAADF-STEM observations were prepared by crushing; crushed specimens were dispersed in methanol and scooped onto a lacey carbon microgrid. The chemical compositions were measured by energy-dispersive X-ray spectroscopy (EDS) in the STEM. The phase-transformation temperatures were determined by differential scanning calorimetry (DSC) under high-purity Ar gas flow. Low-temperature differential scanning calorimetry (L-DSC; DSC8231, Rigaku, Japan) was conducted at a heating/cooling rate of 5 K/min from room temperature to 500°C, and high-temperature DSC (H-DSC; DSC 404 F3, NETZSCH, Germany) was conducted at 10 K/min to 1250°C. Ex situ powder XRD (MiniFlexII, Rigaku, Japan) was conducted at room temperature with Cu–Kα radiation in the 2*θ* range of 10–100°. In-situ XRD (X’Pert Pro, PANalytical, Nederland) was conducted at 350°C and 450°C in the 2*θ* range of 32–36°. The resultant profiles were evaluated by fitting a pseudo-Voigt function and the lattice parameters were determined by the least-squares method.

**Table 2.** Transformation temperatures and reactions from DSC heating curves in Figure 3

Nominal composition (at.% Al)	Temperature (°C)	Reaction
69.0	1150–1167	*ζ → η + ε →* L*η + *L *→* L
70.0	1152–1167	*η → η + *L *→* L
70.4	387	*η*” → *η*
	1150–1169	*η → η + *L *→* L
70.8	250–343	*η*^m^ → *η*^m^ + *η*” → *η*”
	361	*η*” → *η*
	1147–1168	*η → η + *L *→* L
71.0	250–343	*η*^m^ → *η*^m^ + *η*” → *η*”
	352	*η*” → *η*
	1146–1165	*η → η + *L *→* L
71.2	343	*η*^m^ → *η* +_ _*η*”
	1150–1165	*η → η + *L *→* L
71.4	343	*η*^m^ → *η* +_ _*η*”
	1135	*η → η* *+ θ*
	1146–1165	*η → η + *L *→* L
71.5	312	*η*’ → *η*^m^ + *η*”’
	331	*η*^m^ + *η*”’→ *η*
	343	*η*^m^ → *η* + *η*”
	1110	*η → η* *+ θ*
	1145–1162	*θ → η + *L *→* L
71.6	312	*η*’ → *η*^m^ + *η*”’
	331	*η*^m^ + *η*”’ → *η*
	1087	*η → η* *+ θ*
	1146–1163	*θ → η + *L *→* L
71.8	312	*η*’ → *η*^m^ + *η*”’
	331	*η*^m^ + *η*”’ → *η*
	1053	*η → η* *+ θ*
	1144–1162	*θ → η + *L *→* L
72.0	312	*η*’ → *η*^m^ + *η*”’
	312–331	*η*^m^ + *η*”’ → *η*”’
	~350	*η*”’ → *η*
	1027	*η → η* *+ θ*
	1146–1166	*θ → η + *L *→* L
72.2	312	*η*’ → *η*^m^ + *η*”’
	312–331	*η*^m^ + *η*”’ → *η*”’
	~360	*η*”’ → *η*
	992	*η → η* *+ θ*
	1146–1161	*θ → η + *L *→* L
72.4	312	*η*’ → *η*^m^ + *η*”’
	312–331	*η*^m^ + *η*”’ → *η*”’
	~380	*η*”’ → *η*
	941	*η → η* *+ θ*
	1146–1161	*θ → η + *L *→* L
72.6	312	*η*’ → *η*^m^ + *η*”’
	312–331	*η*^m^ + *η*”’ → *η*”’
	~390	*η*”’ → *η*
	900	*η → η* *+ θ*
	1146–1161	*θ → η + *L *→* L
73.0	~405	*η*”’ → *η*
	1146–1161	*θ → η + *L *→* L

**Figure 2.**
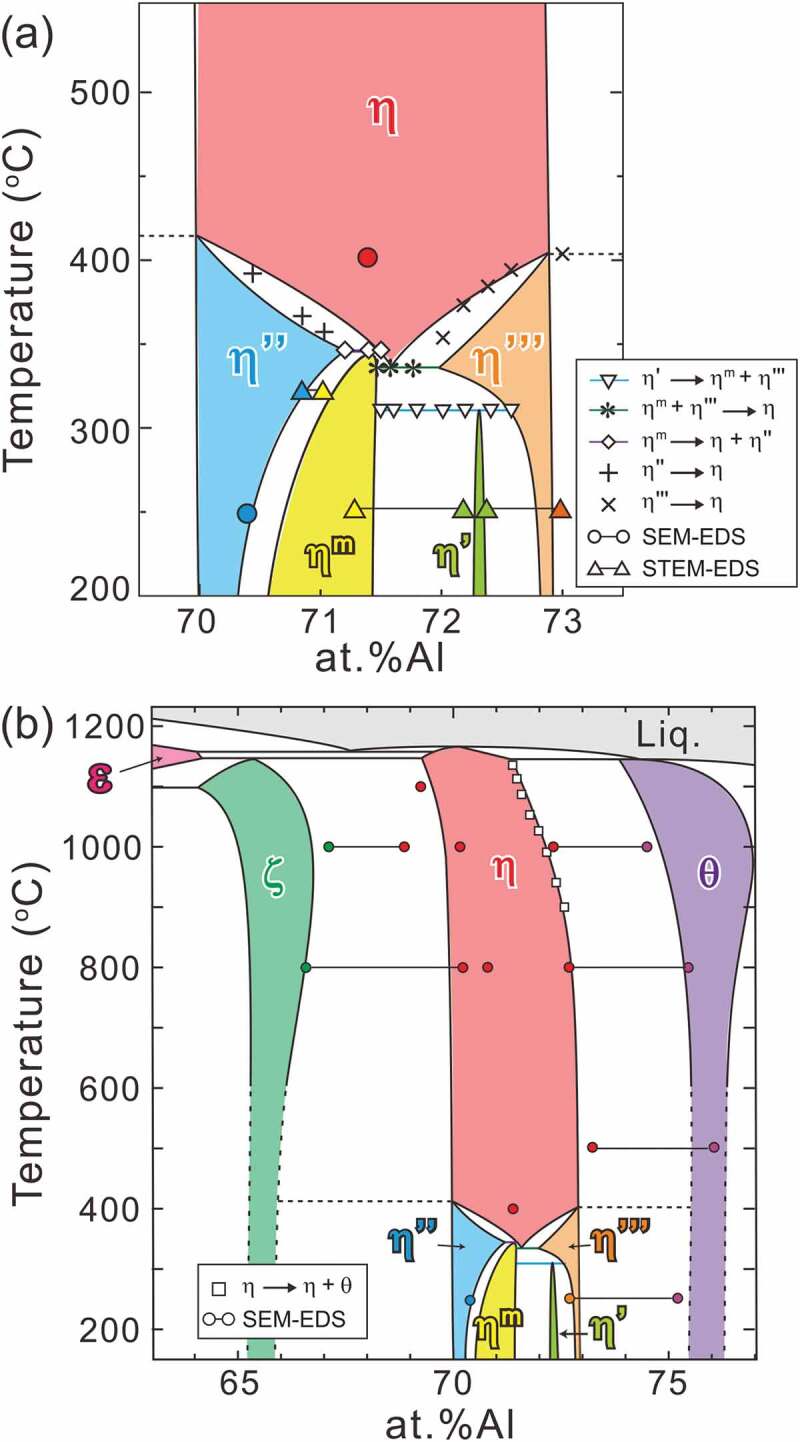
Experimentally determined phase diagram: (a) enlarged low-temperature section and (b) overall view

An outline of our experiments is as follows. DSC measurements were performed to locate the phase-transformation temperatures, EDS analyses were performed to obtain the tie lines between the equilibrating phases, and crystal structure determination by XRD, TEM and STEM was performed to label the observed phases. The phase diagram shown in Figure 2 is established by wrapping up all the results with overall consistency. Unless otherwise stated, the temperatures in the figures, tables and text represent the equilibrated temperatures and not the in-situ experimental temperature.

**Figure 3.**
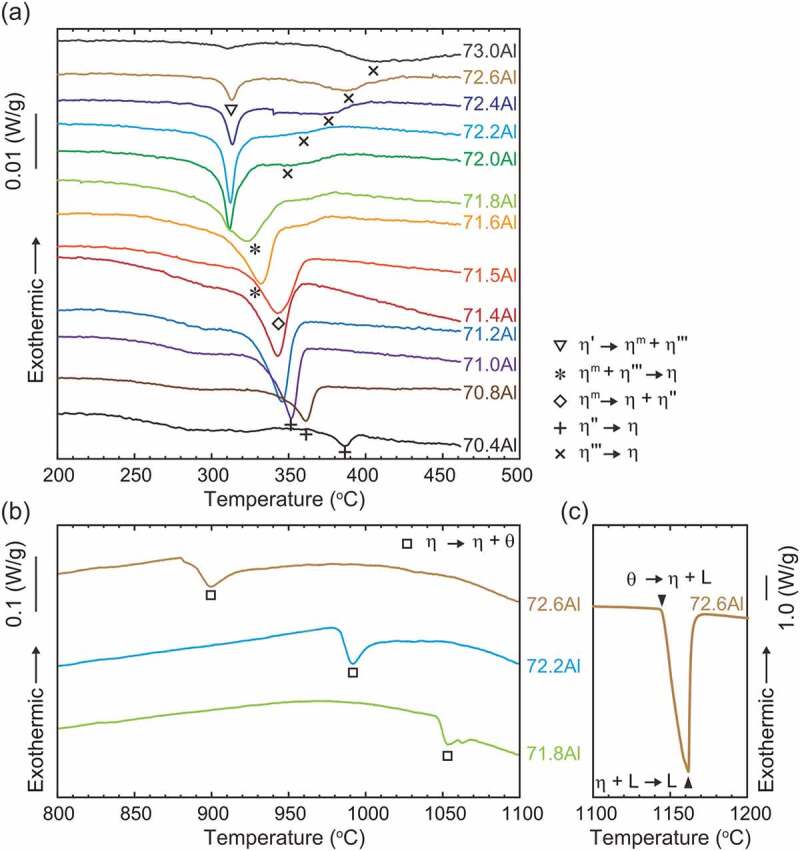
(a) L-DSC and (b and c) H-DSC heating curves for samples equilibrated at 250°C. The vertical scale is shown next to the figure. The temperatures and reactions indicated by various symbols are listed in Table 2

## 3. Results

### 3.1. Thermal analysis

Figure 3(a–c) presents the L-DSC and H-DSC heating curves for specimens equilibrated at 250°C, respectively. The transformation temperatures and reactions evaluated from these DSC results are tabulated in Table 2. In the L-DSC curves (Figure 3(a)), sharp endothermic peaks at ~312°C (marked with ▽) and ~343°C (marked with ◇) are clearly visible for the 72.0–72.6 at.%Al and 71.2–71.5 at.%Al specimens, respectively. These peaks correspond to the peritectoid reactions of *η*’ → *η*^m^ + *η*”’ and *η*^m^ → *η *+ *η*” (low- and high-temperature phases are placed on the left and right terms, respectively, in the reactions in this paper). Furthermore, as described later, the *η* phase is no longer stable below 331°C. According to the Gibbs phase law, another invariant reaction should exist; dull peaks at an intermittent temperature (~331°C, marked with *) in the 71.6 at.%Al and 71.8 at.%Al specimens are assumed to be responsible for the eutectoid reaction of *η*^m^ + *η*”’ → *η*, where the partial superposition of the neighboring two peritectoid reactions may result in peak blurring. As discussed in detail in Section 4.1, the small humps at ~350–400°C (marked with + and × for the Al-poor and Al-rich sides, respectively) are considered to result from the phase transformations of *η*” → *η* and *η*”’ → *η* bypassing their narrow two-phase regions. All data points are indicated in the phase diagram of the low-temperature section in Figure 2(a). In the H-DSC heating curves (Figure 3(b,c)), endothermic peaks corresponding to the reaction *η* → *η* + *θ* (marked with □) are visible. The reaction temperature shifts downward monotonously with Al concentration, which is consistent with the previously reported Fe–Al phase diagram [13–16].

### 3.2. Crystallographic property

**Figure 4.**
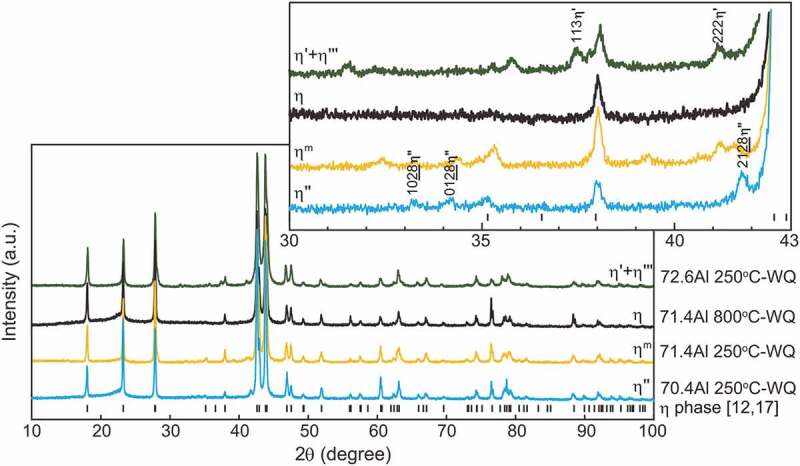
Typical powder XRD patterns obtained from specimens that form a single phase except for the 72.6 at.%Al specimen equilibrated at 250°C. The reference peak positions of the *η* phase with the reported lattice dimension [12] and site occupancy [17] are appended with black bars. Miller indices indexed with the ordered *η*’ and *η*” phases [18,19] are also given in the enlarged graph

**Figure 5.**
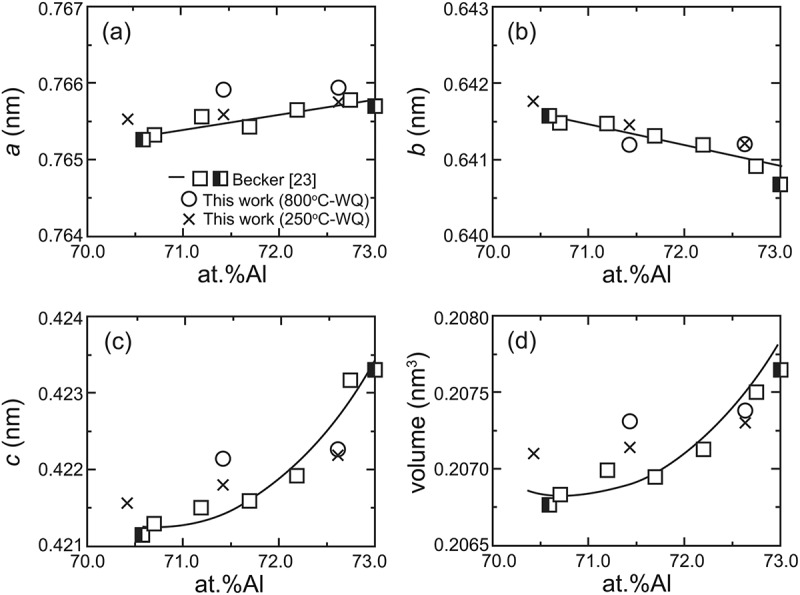
Composition-dependent changes in lattice parameters and volumes

Typical ex-situ powder XRD patterns are presented in Figure 4 together with reference reflection positions of the *η* phase with the reported lattice dimensions [12] and site occupancy [17]. Because the solubility range of the *η*’ phase is narrow, a *η*’ single phase could not be obtained. All specimens, except for the 71.4 at.%Al specimen equilibrated at 800°C, exhibit superlattice reflections because of the higher-order ordering of Al and Fe atoms in the *c*-axis chain sites as suggested in Zienert’s and Rank’s XRD studies [21,22], in addition to fundamental reflections from the *η* phase, especially between 30° and 43° in 2*θ* (see inset in Figure 4). Superlattice reflections from the *η*’ and *η*” phases can be indexed consistently with the crystal structures reported by Okamoto et al. [18,19]. Lattice dimensions that correspond to lattice parameters of the parent orthorhombic crystal structure of the *η* phase [12] were calculated for higher-ordered phases (*η*’, *η*”, *η*”’ and *η*^m^) and are shown in Table 3 and Figure 5 as a function of Al content.

**Table 3.** Lattice parameters and volumes calculated using Burkhardt’s unit cell [12]

	Lattice parameter (nm)			Volume (nm^3^)
Specimen	*a*	*b*	*c*	
Fe-70.4Al 250°C-WQ	0.7655 ± 0.0002	0.6417 ± 0.0002	0.4216 ± 0.0001	0.2071 ± 0.0002
Fe-71.4Al 250°C-WQ	0.7656 ± 0.0002	0.6414 ± 0.0001	0.4218 ± 0.0001	0.2071 ± 0.0002
Fe-71.4Al 800°C-WQ	0.7659 ± 0.0002	0.6412 ± 0.0002	0.4221 ± 0.0001	0.2073 ± 0.0002
Fe-72.6Al 250°C-WQ	0.7658 ± 0.0004	0.6412 ± 0.0003	0.4222 ± 0.0002	0.2073 ± 0.0003
Fe-72.6Al 800°C-WQ	0.7659 ± 0.0003	0.6412 ± 0.0002	0.4223 ± 0.0002	0.2074 ± 0.0002

As shown in Figure 5(a–c), changes in the orthorhombic lattice parameters with Al concentration lie roughly on a single master curve, indicating that the robust FeAl_2_ framework structure dictates the lattice size regardless of how ordering of Al and Fe atoms occurs in the *c*-axis chains. Besides, remarkable composition dependence is only visible in the *c*–axis dimension (Figure 5(c)), which results in the same trend in the unit cell volume (Figure 5(d)). This trend suggests that the increasing Al/Fe ratio in the *c*-axis chain sites with Al content essentially results in the *c*–axis elongation regardless of the atomic ordering therein.

**Figure 6.**
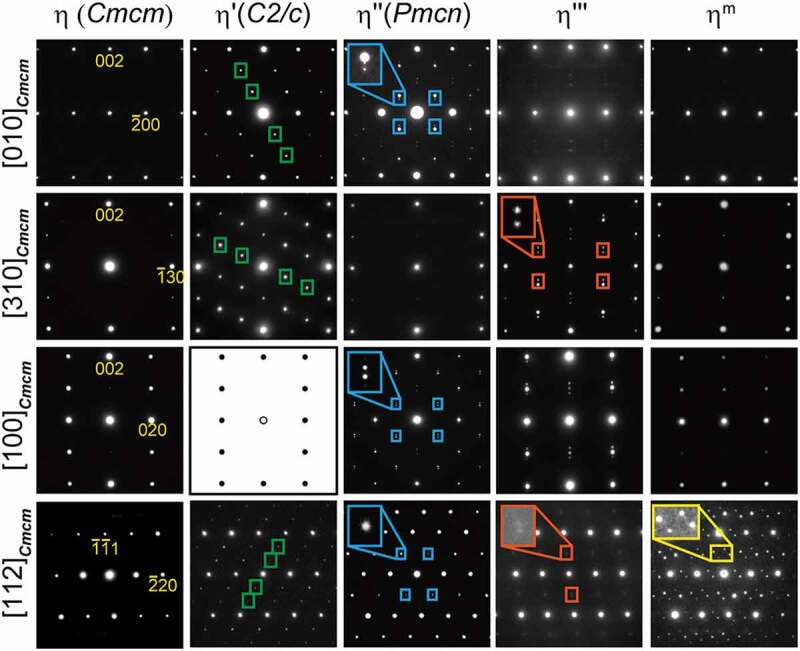
SAED patterns obtained from the *η* phase and its higher-ordered *η*’, *η*”, *η*”’, and *η*^m^ phases with several beam incidences. Superlattice reflections are highlighted with colored rectangles

Figure 6 presents a series of selected-area electron diffraction (SAED) patterns obtained from the *η* phase and its higher-ordered phases with various incidences. All higher-ordered phases exhibit superlattice reflections at particular positions as highlighted with colored rectangles, in addition to fundamental reflections from the *η* structure. As reported by Okamoto et al. [18], the *η*’ phase shows superlattice reflections at positions that divide the distance between the 000 and some fundamental spots by three as indicated by the green rectangles. Unlike in the *η*’ phase, superlattice reflections of the *η*” phase, on the other hand, do not occur at mid positions between the 000 and some fundamental spots but split into two spots around these mid positions along the c* direction (* denotes reciprocal vector) as indicated by the blue rectangles. Okamoto et al. [19] found that this splitting originates from the occurrence of structural and compositional modulations in the higher-ordered structure. Superlattice reflections of the *η*”’ phase split into two spots along the c* direction similarly in the *η*” phase as indicated by the orange rectangles. This is consistent with the report by Becker et al. [17]. Although incidences of diffraction positions around which splitting occurs and their splitting distance along the c* direction differ from each other in the *η*” and *η*”’ phases, the occurrence of splitting of superlattice reflections strongly indicates that the *η*”’ phase possesses a higher-ordered crystal structure that contains structural and compositional modulations like the *η*” phase. Superlattice reflections of the *η*^m^ phase appear in a more complicated manner along the non-c* direction(s), as indicated by the yellow rectangles. The *η*^m^ phase is thus considered to have a higher-ordered crystal structure that contains structural and compositional modulations that differ from those of the *η*” and *η*”’ phases. The local atomic arrangements of the *η*”’ and *η*^m^ phases will be reported elsewhere. The occurrence of splitting of the superlattice reflections allows for the four higher-ordered phases to be classified into two; one with structural and compositional modulations (*η”, η*”’, and *η*^m^ phases) and the other without modulation (*η*’ phase).

### 3.3. Microstructural observation and chemical analysis

**Figure 7.**
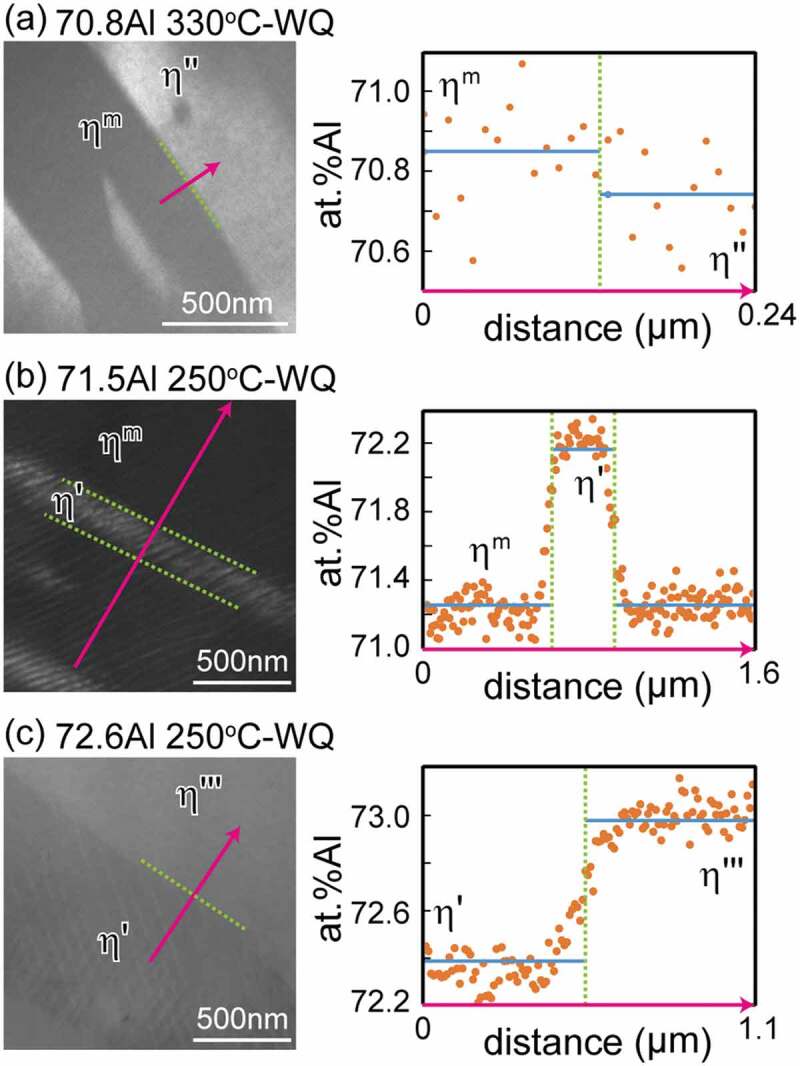
Typical dark-field TEM images of two-phase microstructures and corresponding chemical profiles obtained by STEM-EDS for (a) 70.8 at.%Al homogenized at 330°C (b) 71.5 at.%Al homogenized at 250°C, and (c) 72.6 at.%Al homogenized at 250°C. TEM image in (a) was taken using the 3$\overset{ˉ}{1}$1 superlattice reflection with the incidence [11$\overset{ˉ}{2}$]*_Cmcm_*. TEM image in (b) was taken using the $\overset{ˉ}{3}$30 superlattice reflection with the incidence [112]*_Cmcm_*. TEM image in (c) was taken using the 11$\overset{ˉ}{2}$ superlattice reflection with the incidence [111]*_Cmcm_.*

Figure 7 presents typical dark-field TEM images of two-phase microstructures that are composed of any *η* phase and/or its higher-ordered phases and chemical profiles along the arrows. The constituent phases were identified by SAED. The differences in compositions among these phases are very small, generally less than 1 at.%Al, and each phase precipitates finely with particular orientation relationships. The average equilibrium compositions of each phase were evaluated as indicated by the blue solid lines and listed in Table 4. The tie lines are provided in the low-temperature section of the phase diagram of Figure 2(a). According to Becker et al. [17], the *η* phase has a relatively wide solubility range of 70.6 at.%Al to 73.0 at.%Al at 750°C in response to the varying occupancy ratio of the Al/Fe atoms in the *c*-axis chain. Okamoto et al. [19] reported a certain solubility range of the *η*” phase and highlighted the possibility to be achieved by changes in the modulation periodicity. Because the *η*”’ phase is known to have a modulated structure like in the *η*” phase [17] and the *η*^m^ phase is expected to have a similar crystallographic feature as can be inferred from Figure 6, they may have a certain extent of solubility. The *η*’ phase, on the other hand, possesses a crystal structure that does not contain structural and chemical modulation [18,23], and thus exhibits a limited solubility, because only varying site occupancy in the *c*-axis chain is allowed.

**Table 4.** Nominal and equilibrium compositions of alloys decomposed into dual phases

				Equilibrium composition (at.% Al)	
Nominalcomposition (at.% Al)	Annealing temperature (°C)	Annealing time (day)	Phase 1/Phase 2	Phase 1	Phase 2
69.0^a^	300	30	*ζ*/*η*”	67.11	70.36
69.0^a^	800	3	*ζ*/*η*	66.59	70.27
69.0^a^	1000	1	*ζ*/*η*	66.00	68.86
70.8^b^	330	12	*η*”/*η*^m^	70.74	70.85
71.5^b^	250	60	*η*^m^/*η*’	71.26	72.15
72.6^b^	250	60	*η*’/*η*”’	72.39	72.98
73.0^a^	250	60	*η*”’/*θ* (/*η*’)	72.69	75.40
73.0^a^	330	12	*η*”’/*θ*	72.55	75.25
73.0^a^	500	10	*η*/*θ*	73.15	76.10
73.0^a^	800	0.5	*η*/*θ*	72.20	75.21
73.0^a^	1000	0.5	*η*/*θ*	72.32	74.49

^a^Determined by SEM-EDS.

^b^Determined by STEM-EDS.

### 3.4. Phase diagram

The phase diagram constructed in this study is shown in Figure 2(a,b). The *η*” and *η*”’ phases are in equilibrium with the *ζ* and *θ* phases, respectively. The *η* phase is no longer stable at a low temperature and terminates at 331°C with ~71.5 at.%Al. This composition agrees well with the stoichiometric composition (71.4 at.%Al) of *η*-Fe_2_Al_5_. The low-temperature phase diagram, which is shown in Figure 2(a), is complicated. Three invariant reactions exist: *η*’ → *η*^m^ + *η*”’ at 312°C, *η*^m^ + *η*”’ → *η* at 331°C, and *η*^m^ → *η* + *η*” at 343°C. The higher-ordered phases can be classified into two types in terms of their phase equilibria: the *η*” and *η*”’ phases that are in equilibrium with the neighboring phases of the *ζ* phase below ~415°C and the *θ* phase below ~405°C, respectively, and the *η*’ and *η*^m^ phases that are in equilibrium with the *η* or its higher-ordered phases only at low temperature. On the other hand, they can also be classified into three in terms of the solubility range. The *η* phase exhibits a relatively large solubility range at elevated temperatures by changing the occupancy ratio of Al/Fe atoms in the *c*-axis chain sites. The *η”, η*”’ and *η*^m^ phases also host a relatively large solubility range by changing the periodicity of the chemical/structural modulations. The *η*’ phase exhibits a limited solubility range within its limited stable temperature range because of a lack of chemical/structural modulations in the structure.

## 4. Discussion

### 4.1. *Determination of* η*/*η*” and* η*/*η*”’ phase boundaries*

**Figure 8.**
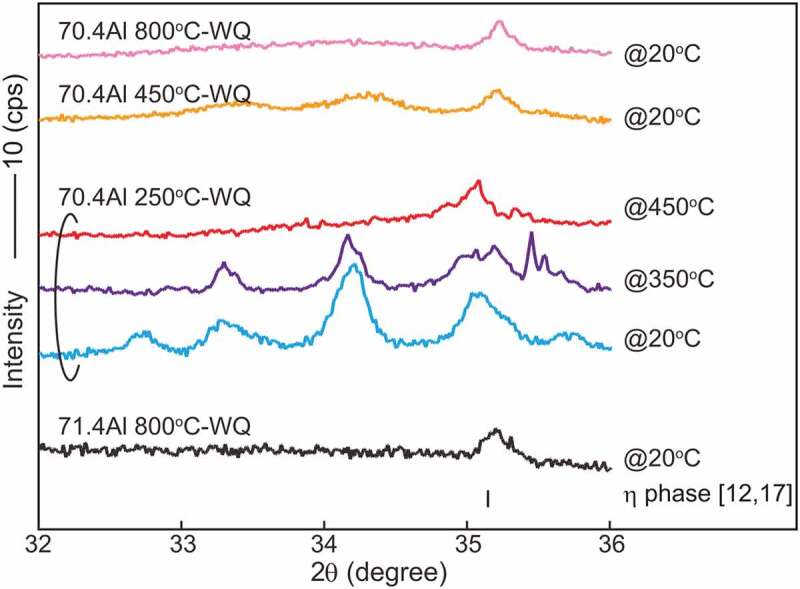
Detailed in situ and ex situ powder XRD patterns. Scan speed was 0.2°/h

The location of the *η*/*η*” and *η*/*η*”’ phase boundaries remains controversial and indeed we did not capture any signs in the H-DSC curves above 500°C. While Becker has mentioned that rapid quenching may be crucial to suppress ordering into the *η*” and *η*”’ phases [17], Okamoto has reported that the *η*” phase forms in specimens quenched from 1000°C [19]. To locate these phase boundaries and to examine quenchability of the *η* phase, we performed detailed investigations for the 70.4 at.%Al specimen equilibrated at various temperatures by integrating in- and ex-situ XRD, L-DSC, and SAED. Figure 8 shows detailed in- and ex-situ XRD profiles in the 2*θ* range of 32–36°. A series of in-situ XRD profiles for a 70.4 at.%Al specimen equilibrated at 250°C shows a remarkable change between 350°C and 450°C; multiple superlattice reflections that are visible at 20°C and 350°C disappear and only one reflection remains at 450°C. Because the angle of this residual reflection is close to that of the *η* phase, it can be deduced that the small hump in the L-DSC curves (see Figure 3(a); marked with +) is attributed to the *η*/*η*” phase transformation. From similarities in the crystal structure and L-DSC curves, it can also be deduced that the *η*/*η*”’ phase transformation occurs at temperatures marked with × in Figure 3(a).

**Figure 9.**
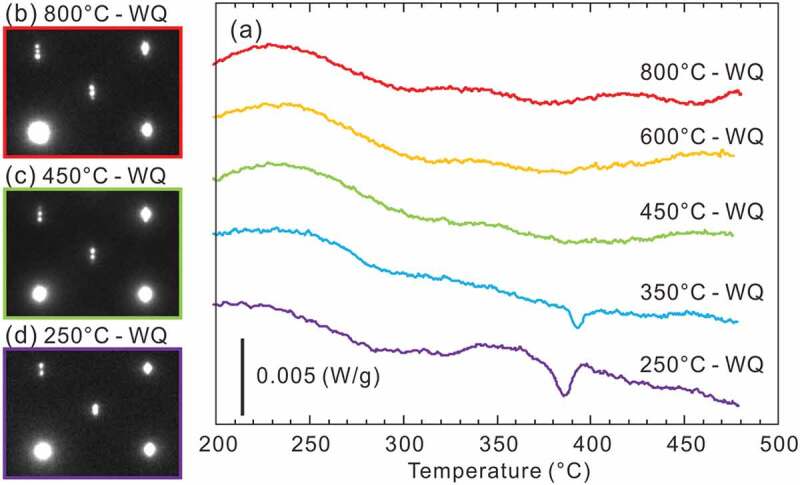
Quenching temperature dependence of L-DSC curve and SAED pattern for a 70.4 at.%Al specimen. To avoid possible error in composition among samples, these data were assembled through one specimen by repeatedly equilibrating at displayed temperatures (followed by water quenching) in order from the lowest to the highest temperatures

Meanwhile, ex-situ XRD profiles for specimens quenched from 450°C and 800°C exhibit diffuse reflections together with a sharp reflection of the *η* phase. Because the angle of these diffuse reflections is close to those of the *η*” phase (blue profile), it is reasonable that quenching of the *η* phase fails and some degree of atomic ordering to the *η*” structure develops during quenching. The thermal stability and crystallographic feature of this un-quenched *η* phase are accounted for from the L-DSC curves and SAED patterns summarized in Figure 9. Note that these data were assembled through one specimen by repeated equilibration at the displayed temperatures (followed by water quenching) in order from the lowest to the highest temperatures to avoid possible error in composition among samples. Of particular interest is that the small hump at ~400°C that appears when homogenized below this temperature becomes invisible for samples homogenized above this temperature. Nevertheless, their SAED patterns show no significant difference, which supports that some extent of partial ordering develops as deduced from the ex-situ XRD profiles. From Figures 8 and 9, we can elicit the following conclusion on thermal stability and quenchability of the *η* phase. It is found to be difficult to quench the *η* phase through conventional water quenching, as originally suggested by Becker et al. [17]. Even for a very short time during quenching, some degree of atomic ordering develops. This trend could become more pronounced as the composition deviates from the stoichiometry of 71.4 at.%Al. The small hump in the L-DSC curves is most likely identical to the *η*/*η*” phase-transformation temperature where the two-phase region cannot be resolved because of the narrowness. It remains less reasonable that the small hump is missing for samples that were equilibrated above this temperature even though they would involve *η*” ordering to some extent in degree and/or range after quenching. We consider that the difference in inter- and intra-chain diffusivity in the *c*-axis chain plays a key role in developing the long-range *η*” ordering. In what follows, the detailed atomic structures of specimens that were quenched from temperatures below and above the *η*/*η*” phase-transformation temperature are presented.

### 4.2. *Refinement of crystal structure of* η*” phase with finite antiphase boundary width model*

According to Okamoto et al. [19], the *η*” phase forms a long-period-ordered superlattice structure (space group *Pmcn*) that consists of motif slabs based on the parent orthorhombic lattice stacked along the *c*-axis with periodically introduced atomic slabs that yield structural and chemical modifications. Of particular interest is that the *η*” phase can accommodate a certain solubility range by changing the periodicity of such modulation because the atomic species and ratio of the Al2-equivalent sites (100% Al) in the atomic slab that yields structural and chemical modifications are different from those (50% Fe + 50% Al) in the motif slabs [19]. However, it remains questionable whether such an abrupt change in composition occurs at the atomic slab boundary in light of the thermodynamic (local) equilibrium. Thermodynamic equilibrium dictates a gradual change in composition [24] and accordingly modifies the geometrical feature of the chemical composition variation. To capture the essential feature of structural/chemical modifications, we performed HAADF-STEM observations for the *η*” phase of a 70.4 at.%Al specimen equilibrated below and above the *η*/*η*” phase-transformation temperature and exploited the geometrical features by performing geometrical phase analysis.

**Figure 10.**
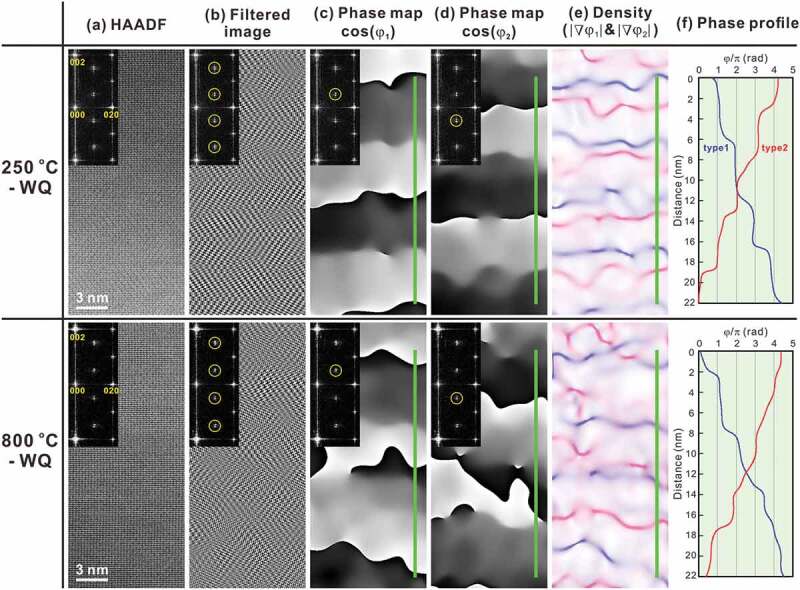
(a) [100]-projected HAADF-STEM images of *η*” phase obtained from 70.4 at.%Al specimens homogenized at 250°C (upper panels) and 800°C (lower panels). Respective FFT images are also appended. (b) Filtered images of (a) through iFFT process using FFT spots highlighted by yellow circles. (c and d) Corresponding geometrical phase (*φ*_1_ and *φ*_2_) images presented in the form of cosine, where *φ*_1_ and *φ*_2_ are assigned as FFT spots highlighted by yellow circles. (e) Density (|▽*φ*_1_|and|▽*φ*_2_|) maps, where|▽*φ*_1_|and|▽*φ*_2_|are scaled with blue and red, respectively. (f) Geometrical phase profiles of *φ*_1_ and *φ*_2_ along green lines given in (c–e)

[100]-projected HAADF images of the specimens equilibrated at 250°C and 800°C are presented in Figure 10(a). Their fast-Fourier transform (FFT) patterns, inserted in Figure 10(a), show the superlattice spots at the mid positions between the 0 2 *n m* and 0 2(*n* ± 1) *m* ± 1 fundamental spots (*n* and *m* are integer) split along the c* direction, which indicates that these HAADF images indeed capture the microstructural feature that reproduces the SAED pattern of Figures 6 and 9. To exploit the microstructural feature that causes the split superlattice spots, an inverse FFT (iFFT) was processed after masking areas in the FFT images except for the superlattice spots (see insets in Figure 10(b)), and reconstructed images are presented in Figure 10(b). The image of the specimens equilibrated at 250°C exhibits a tweed-like texture, which is most likely identical to the stacked motif slab. In the counterpart quenched from 800°C, this texture is less discernible but maze-like domains form, which indicates that the short-range ordering of the *η*” structure could be developed during quenching. The inherent geometrical feature can be manifested by performing geometrical phase analysis; we herein regard the HAADF images as being decomposable into interference images, the fringe pitches of which are identical to the reciprocals of the distance between the 0 0 0 and intended spots in the FFT space. To capture the geometrical features that originate from the split superlattice spots, we selected the 0 1 1/2 and 0 1 − 1/2 spots (see insets of Figure 10(c,d), respectively) and their iFFT-processed interference images were reconstructed into the geometrical phase (*φ*_1_ and *φ*_2_) maps. These phase maps are presented as cos(*φ*_1_) and cos(*φ*_2_) in Figure 10(c,d), respectively. In the image of the specimen quenched from 250°C, changes in *φ*_1_ and *φ*_2_ are highly localized in planar regions that are nearly parallel to the (001)*_Cmcm_* plane and these regions are introduced alternately with a nearly constant interval. These features are less discernible in the image of 800°C, and the orientation propensity is significantly violated. The split superlattice patterns in the FFT images at both temperatures are attributed to this stacking periodicity along the *c*-axis. The density map of the geometrical phase gradient, i.e. |▽*φ*_1_| and |▽*φ*_2_|, shown in Figure 10(e) is helpful to locate the planar defects, where the magnitude of the phase gradient is described by the colored contrast. Again, changes in *φ*_1_ and *φ*_2_ are highly localized at the planar defects, and two types of defects stack alternately nearly along the [001]*_Cmcm_* direction when quenched from 250°C. Hence, the image can capture the essential features of the long-period-ordered superlattice structure of the *η*” phase. When quenched from 800°C, in contrast, localization of the phase changes is somewhat diminished and percolates into the motif regions, and the alternative arrangement of these defects is partly broken. Finally, phase profiles along the green lines in Figure 10(c–e) are provided in Figure 10(f). In the specimen quenched from 250°C, *φ*_1_ and *φ*_2_ takes discrete values in the motif slabs, and the change in *φ*_1_ and *φ*_2_ between adjacent motif slabs is π, localized at the planar defects. Taking this constant phase shift and wavy morphology into account, it is concluded that the planar defects are antiphase boundaries (APBs). In the specimen quenched from 800°C, the changes in *φ*_1_ and *φ*_2_ are less discretized, which suggests a thickening of the APBs. The split distances Δ* of the superlattice spots are ~1/16.3 of the reciprocal lattice spacing of the (001) plane c* in both observations, which corresponds to the APB periodicity of ~6.9 nm in real space. This value is within reason compared with the real space observations, as shown in Figure 10.

**Figure 11.**
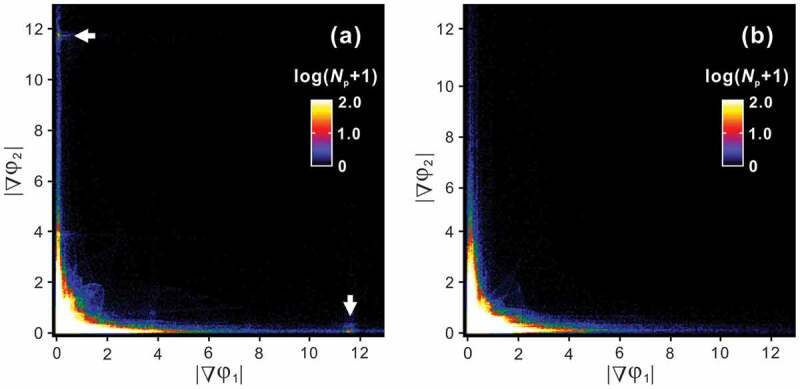
Bivariate histograms with variables of |▽*φ*_1_| and |▽*φ*_2_| obtained from (a) upper and (b) lower panels of Figure 10(e) where *N*_p_ is the number of pixels

Figure 10 clarifies that a remarkable difference in the atomic structures of the equilibrated and non-equilibrated *η*” phase is the morphology of APB domain structure. This contrast should be responsible for the appearance of a small hump in the L-DSC curves (Figure 9(a)). We therefore exploit this microstructural feature and discuss the origin of the small hump. We provide a statistical presentation of the geometrical phase images of Figure 10(c,d); the bivariate histograms with variables of |▽*φ*_1_| and |▽*φ*_2_| are presented in Figure 11(a,b) for 250°C and 800°C, respectively. A remarkable difference is observed at (|▽*φ*_1_|, |▽*φ*_2_|) = (0, ~12) and (~12, 0), where bright peaks are visible only in the specimen quenched from 250°C. This localization means that a certain amount of APBs aligns precisely parallel to the (001)*_Cmcm_* plane. This orientation preference suggests that some (chemical and/or physical) interactions across the *c*-axis chains, namely, inter-chain interactions play an important role in forming the (001)*_Cmcm_*-oriented APBs, which is presumably the most energetically favorable orientation. The latent heat of the small hump may be consumed mainly to escape this lock-in state. At this temperature, atomic diffusion within every *c*-axis chain could prevail, which results in a decoupling of the interchain interactions and thus the *η*/*η*” phase transformation takes place. The phase quenched from 800°C develops only short-range *η*” ordering but the APBs are unlocked in the (001)*_Cmcm_* plane, thus that the small hump disappears. On the basis of the similarity in highly ordered long-period structures of the *η*” and *η*”’ phases, the small hump in the L-DSC curves for the *η*”’ phase is assumed to have the same origin as that of the *η*” phase.

**Figure 12.**
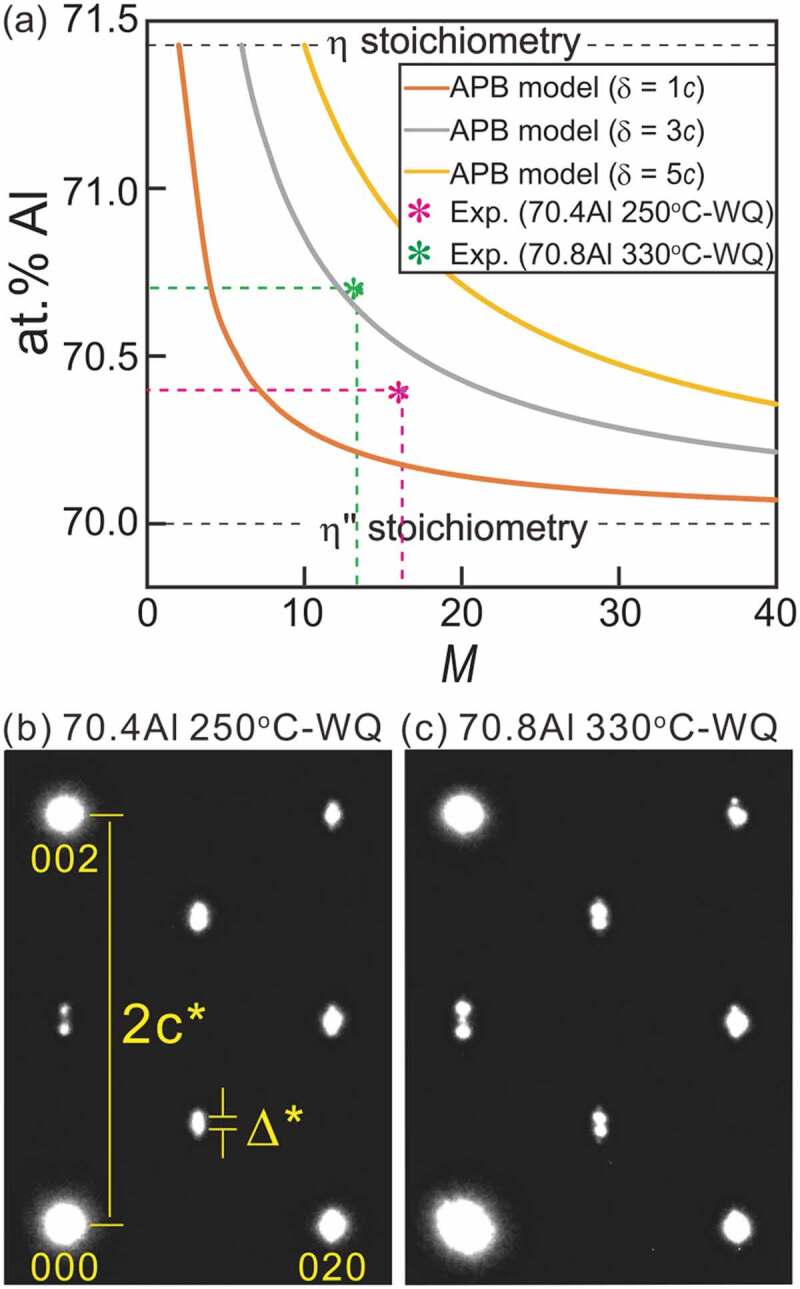
(a) Nominal composition of *η*” phase as a function of number of stacking o*δ*rthorhombic *η* units *M* calculated based on APB model with different widths of *δ* = 1 *c*, 3 *c*, and 5 *c*. SAED patterns taken from *η*” phase in (b) a 70.4 at.%Al specimen homogenized at 250°C and (c) a 70.8 at.%Al specimen homogenized at 330°C

From the information obtained here, it can be deduced that the APBs are accompanied by a structural (chemical) imperfection and finite distribution width. These features are intrinsically inseparable for thermal APBs. The chemical composition of the antiphase domains (APDs) and APBs should be different and the difference is ideally dictated by the common tangent law for the Gibbs free energy curves of the APD (herein *η*”) and APB (*η*) structures [24]. Concomitantly, a discrete change in chemical composition is energetically unfavorable; thus, thermal APBs have a finite width to satisfy the local thermodynamic equilibrium. We evaluate the change in nominal composition of the *η*” phase as functions of the overall long-period cell length that is normalized by the orthorhombic *η* unit (*M*) and the APB width *δ*. Figure 12(a) and Table 5 shows the nominal composition of the *η*” phase as a function of *M* with the APBs (*η*) with various widths of *δ* = 1*c*, 3*c*, and 5*c*. The composition of the APB (*η*) is set to a stoichiometric composition of 71.4 at.%Al with an Fe/Al mixed occupation in the Al2 and Al3 sites reported by Becker et al. [17] and discrete changes in the composition at the APB(*η*)/APD(*η*”) boundary are assumed. The nominal composition, as a reasonable consequence, terminates at the *η*-Fe_2_Al_5_ stoichiometry when *δ*/*c* = *M* and converges toward the *η*”-Fe_3_Al_7_ stoichiometry toward *M* → ∞. The SAED patterns of the *η*” phase for 70.4 at.%Al annealed at 250°C and 70.8 at.%Al at 330°C are presented in Figure 12(b,c), where the latter alloy consists of the *η*” phase with 70.7 at.%Al and the *η*^m^ phase with 70.9 at.%Al (see Figure 7(c)). The ratio of Δ*/c* (=*M*^−1^) is approximately 1/16.3 and 1/13.5, respectively; these values are plotted in Figure 12(a). As a rough approximation, we can estimate an APB width *δ* to be ~2.8*c*, i.e. ~1.2 nm. This value is within reason compared with reported *δ* values in different ordered alloy systems [25–27].

**Table 5.** Nominal composition of *η*” phase calculated based on APB models with different width *δ* for various *M* values

	*M*	2	4	6	8	10	12	14	16
Composition (at.% Al)	*δ* = 1 *c*	71.43	70.71	70.48	70.36	70.29	70.24	70.20	70.18
	*δ* = 3 *c*			71.43	71.07	70.86	70.71	70.61	70.54
	*δ* = 5 *c*					71.43	71.19	71.02	70.89

## 5. Conclusions

In this study, we determined the phase equilibria among the *η*-Fe_2_Al_5_ phase and its peripheral higher-ordered phases through intensive thermal, crystallographic, chemical, and atomistic investigations. Although the *η* phase has been considered stable over the temperature range below the melting point, it turned out to be unstable below 331°C, and decomposed into the *η*^m^ and *η*’ phases. The *η*” and *η*”’ phases are in equilibrium with the neighboring *ζ*-FeAl_2_ and *θ*-Fe_4_Al_13_ phases, respectively. The crystallographic features of the higher-ordered phases can be classified into the following two types: one with structural and compositional modulations that involve APBs (*η”, η*”’, and *η*^m^ phases) and the other without modulation (*η*’ phase). APBs with a finite thickness were shown to play a key role in tolerating certain solubility ranges in the *η”, η*”’, and *η*^m^ phases. The phase diagram incorporates phase equilibria among the *η*-Fe_2_Al_5_ phase and its higher-ordered phases of the *η*’, *η*”, *η*”’, and *η*^m^ phases, which have not been accounted for in any existing phase diagrams. This reassessment provides a fundamental and broader impact on material design guidelines for the vast array of practical Fe-based alloy systems.
